# Tyrosine Sulfation of Human Trypsin Steers S2’ Subsite Selectivity towards Basic Amino Acids

**DOI:** 10.1371/journal.pone.0102063

**Published:** 2014-07-10

**Authors:** András Szabó, Moh’d A. Salameh, Maren Ludwig, Evette S. Radisky, Miklós Sahin-Tóth

**Affiliations:** 1 Department of Molecular and Cell Biology, Boston University Henry M. Goldman School of Dental Medicine, Boston, Massachusetts, United States of America; 2 Department of Cancer Biology, Mayo Clinic Cancer Center, Jacksonville, Florida, United States of America; 3 Pediatric Nutritional Medicine & EKFZ, Technische Universität München (TUM), Munich, Germany; National Research Council of Italy, Italy

## Abstract

Human cationic and anionic trypsins are sulfated on Tyr154, a residue which helps to shape the prime side substrate-binding subsites. Here, we used phage display technology to assess the significance of tyrosine sulfation for the specificity of human trypsins. The prime side residues P1′–P4′ in the binding loop of bovine pancreatic trypsin inhibitor (BPTI) were fully randomized and tight binding inhibitor phages were selected against non-sulfated and sulfated human cationic trypsin. The selection pattern for the two targets differed mostly at the P2′ position, where variants selected against non-sulfated trypsin contained primarily aliphatic residues (Leu, Ile, Met), while variants selected against sulfated trypsin were enriched also for Arg. BPTI variants carrying Arg, Lys, Ile, Leu or Ala at the P2′ position of the binding loop were purified and equilibrium dissociation constants were determined against non-sulfated and sulfated cationic and anionic human trypsins. BPTI variants harboring apolar residues at P2′ exhibited 3–12-fold lower affinity to sulfated trypsin relative to the non-sulfated enzyme, whereas BPTI variants containing basic residues at P2′ had comparable affinity to both trypsin forms. Taken together, the observations demonstrate that the tyrosyl sulfate in human trypsins interacts with the P2′ position of the substrate-like inhibitor and this modification increases P2′ selectivity towards basic side chains.

## Introduction

The human pancreas secretes two trypsinogen isoforms in large quantities, anionic and cationic trypsinogens, which account for more than 95% of total trypsinogen content in the pancreatic juice [1]. The two isoforms are highly similar, with about 90% sequence identity. Human trypsinogens become post-translationally sulfated on Tyr154 by the enzyme tyrosylprotein sulfotransferase 2 (TPST2) in the Golgi apparatus [2], [3]. Sulfation in the pancreas is quantitative, whereas trypsinogen expressed in tumors and possibly other tissues may not be sulfated [4]. Trypsinogen sulfation was first observed by Scheele et al. (1981) who demonstrated radioactive sulfur incorporation into trypsinogens in human pancreatic slices incubated with labeled sodium sulfate [1]. The site of sulfation was first revealed by crystallographic studies on native human cationic trypsin by Gaboriaud et al. (1996) who described the presence of a modification on Tyr154, which was incorrectly identified as phosphorylation [5]. In our more recent studies, we isolated and identified the sulfated tyrosine amino acid from hydrolyzed pancreatic trypsinogens, and demonstrated that incorporation of radioactive sulfur was abolished by mutation of Tyr154 [6]. Other investigators used mass spectrometry to confirm tyrosine sulfation of trypsinogens [4], [7]. In characterizing the sequence requirements for sulfation of Tyr154, we found that Asp153 is the main determinant, and that the common African p.D153H variation in anionic trypsinogen causes loss of tyrosine sulfation [8].

The functional significance of tyrosine sulfation in human trypsinogens has remained uncertain so far. Studies on other tyrosine-sulfated proteins as well as phenotypes of TPST1 and TPST2 knock-out animals indicate that the primary function of tyrosine sulfation is modulation of protein–protein interactions among secreted and/or membrane proteins [2], [3], [9]–[12]. Autoactivation of human cationic trypsinogen was somewhat increased by sulfation but a similar effect was not observed with anionic trypsinogen [6], [8]. Increased trypsinogen autoactivation has been implicated as a pathogenic mechanism in chronic pancreatitis, but a genetic study analyzing human *TPST2* variants found no association with chronic pancreatitis [13]. More detailed comparative analysis of non-sulfated and sulfated anionic trypsins did not reveal any appreciable differences with respect to catalytic activity on a variety of substrates, activation by enteropeptidase, proteolytic stability or cellular expression [8].

In the present study, we used phage display technology and inhibitor binding experiments to compare the prime side substrate specificity of non-sulfated and sulfated trypsins. These studies were motivated by the observation that Tyr154 is located on the prime side of the trypsin substrate binding cleft and appears to form part of the S2′ subsite (Schechter and Berger nomenclature [14]), and thus sulfation may result in altered interactions between human trypsins and their inhibitors and substrates.

## Experimental Procedures

### Amino acid numbering

Bovine pancreatic trypsin inhibitor (BPTI) amino acid residues are numbered starting from the first amino-acid of the 58-amino-acid mature, processed protein [15]. Tyr154 in human cationic trypsinogen is numbered starting from the initiator Met of the primary translation product (pre-trypsinogen). This residue corresponds to Tyr151 in the conventional chymotrypsin numbering.

### Plasmid construction and mutagenesis

The pTrapT7 expression plasmids containing the coding DNA of human cationic and anionic trypsinogens were described previously [16]–[18]. The pPICZ-alpha *Pichia pastoris* expression plasmid containing the coding sequence for BPTI was described earlier [19]. BPTI mutants were created by overlap extension PCR mutagenesis and cloned into the pPICZ-alpha plasmid.

### Expression, refolding and purification of human cationic and anionic trypsinogens

Non-sulfated trypsinogens were expressed in *E. coli* BL21(DE3), re-folded *in vitro* and purified with ecotin affinity chromatography as described [16]–[18], [20], [21]. Sulfated anionic and cationic trypsinogens were isolated from human pancreatic juice with Mono-Q ion exchange chromatography followed by ecotin affinity chromatography, as described previously [6], [22]. Trypsinogen was activated with 14 ng/mL (final concentration) human enteropeptidase (R&D Sytems) in 0.1 M Tris-HCl (pH 8.0) and 1 mM CaCl_2_. Trypsin concentrations were determined with active site titration against ecotin.

### Expression and purification of BPTI variants

BPTI was expressed and purified using protocols similar to those we have described previously [23]. *Pichia pastoris* X-33 transformants were grown for 3 days at 30°C using 500 mL buffered methanol-complex medium (BMMY). BPTI was precipitated from the medium with ammonium sulfate at 95% saturation at 22°C. After centrifugation the protein pellet was dissolved in 100 mL 10 mM Tris-HCl (pH 8.0) and dialyzed against 7 L of 10 mM Tris-HCl (pH 8.0). The dialyzed BPTI was purified on a Q-Sepharose anion exchanger column followed by an affinity chromatography step using immobilized bovine trypsin. The concentration of BPTI mutants was determined with active site titration against human cationic trypsin.

### Selection of inhibitor phages against non-sulfated and sulfated human cationic trypsins

The pComb3H plasmid harboring the coding sequence of BPTI [24] was a kind gift from Jacek Otlewski, University of Wroclaw. A library was designed in which the P1 residue (Lys15 in wild-type BPTI) was mutated to Arg, and the P1′–P4′ residues (Ala16, Arg17, Ile18, and Ile19) and the P19′ residue (Val34) were completely diversified by custom synthesis substituting nucleotide mixtures NNS (N = 25% C, 25% A, 25% G, 25% T; S = 50% G, 50% C) for the diversified codons. The non-amplified library was PCR amplified, cloned into the phage display vector pComb3H+BPTI using restriction enzymes XhoI and SpeI, and transformed into the *E. coli* strain TG1, to yield a cloned library of 2.9×10^7^ transformants (GeneArt Life Technologies). This library size represented approximately 9-fold coverage of the theoretically possible number of encoded amino acid sequence combinations (3.2×10^6^), but represented slightly less than the theoretically possible number of nucleic acid sequence combinations (3.4×10^7^). Sequencing of 90 random clones confirmed the unbiased diversity of the library. Non-sulfated and sulfated human cationic trypsin was immobilized overnight in a Nunc 96-well Maxisorp plate using two wells for each protein and 10 µg protein per well in 100 µL of 0.1 M Tris-HCl (pH 8.0) and 1 mM CaCl_2_ at 4°C. The wells were blocked with 200 µL 5 mg/mL BSA dissolved in TBS (50 mM Tris-HCl (pH 7.5), 150 mM NaCl) for 1 h at 22°C. Two control wells were treated with BSA without trypsin. The wells were rinsed six times with TBS containing 0.1% Tween 20. Phages (∼2×10^7^ particles per well) were added to the wells in TBS containing 0.1% Tween 20 and incubated for 1 h at 22°C. The wells were washed 10 times with TBS, 0.1% Tween 20 and bound phages were eluted with 100 µL 0.2 M glycine-HCl buffer (pH 2.2) per well for 10 min at 22°C. The eluted phage populations from two wells were pooled and neutralized with 20 µL of 1.5 M Tris-HCl (pH 8.8). *E. coli* SS320 (MC 1061 F’) strain (Lucigen) was inoculated in 5 mL SB medium containing 0.36% glucose. At OD_600_ ∼0.5 the *E. coli* cells were infected with 200 µL of the eluted phages. Ten µL of the infected cells were used for phage titration using carbenicillin plates for selection. The remaining infected *E. coli* cells served for phage amplification in the presence of VCSM13 helper phage in 50 mL Super Broth medium with glucose containing 50 µg/mL carbenicillin and kanamycin with overnight shaking at 37°C. The cells were collected by centrifugation; the supernatant containing the amplified phagemid library was precipitated with PEG8000, washed twice with TBS and re-suspended in 400 µL TBS. The amplified phagemid library served as input in the next panning cycle.

### Phage ELISA of the selected library members

Individual clones from the third cycle were tested in phage ELISA. *E. coli* colonies containing the pComb3H-BPTI plasmid were grown overnight in 3 mL LB medium with 100 µg/mL ampicillin at 37°C in the presence of VCSM13 helper phage (10^9^ phages/mL). Cells were separated from the phage-containing supernatant by centrifugation. Non-sulfated and sulfated trypsins (2 µg/well) were immobilized overnight to a Maxisorp plate at 4°C. Wells were blocked with 5 mg/mL BSA dissolved in TBS for 1 h at 22°C. Control wells were also blocked without trypsin treatment. Wells were washed four times with TBS containing 0.1% Tween 20. Aliquots (50 µL) of phage supernatant of individual clones were transferred to the wells and incubated for 1 h at 22°C. Wells were rinsed six times with TBS containing 0.1% Tween 20. Horseradish peroxidase (HRP)-conjugated anti-M13 antibody (1 mg/mL stock, GE Healthcare) was diluted 5000-fold with TBS containing 5 mg/mL BSA and 0.1% Tween 20 and 50 µL was added to each well and incubated for 30 min at 22°C. The wells were rinsed six times with TBS containing 0.1% Tween 20 and twice with TBS. ELISA signal was measured spectrophotometrically at 450 nm in a microplate reader after adding 50 µL of 1-Step Turbo TMB-ELISA HRP substrate (Thermo Scientific), incubating for 10 min and stopping the reaction with 50 µL 2 M sulfuric acid. Phage clones yielding ELISA signals against immobilized trypsin at least 3-fold above the BSA control were selected for sequencing.

### Sequence analysis

DNA minipreps of the pComb3H-BPTI plasmids of individual clones were analyzed by sequencing using the reverse primer 5′-AGC GTT TGC CAT CTT TTC ATA ATC-3′. Clones with unique DNA sequences were aligned and amino acid frequencies at the randomized positions were determined. Where indicated, these frequencies were normalized to the expected codon frequencies in the NNS degenerated set, to eliminate the effects of codon bias. For logo representation of the normalized results an input sequence dataset containing 100 sequences was generated representing the normalized amino acid frequencies at each randomized position [25], [26]. The sequence logos were created by the WebLogo program [27].

### Equilibrium binding assays

Binding of BPTI variants to non-sulfated and sulfated cationic and anionic trypsins were characterized by determining the dissociation constant values in equilibrium (K_D_) as described before [26]. BPTI variants (0–100 pM) and trypsin (50 pM) were incubated in 0.1 M Tris-HCl (pH 8.0), 1 mM CaCl_2_, and 0.05% Tween 20 (final concentrations) for 15 h at 22°C in black 96-well plates in 200 µL volume. Free trypsin concentrations were determined with spectrofluorometry after addition of 5 µL 6 mM Z-Gly-Pro-Arg-AMC and measuring the rate of substrate cleavage using excitation and emission wavelengths of 380 nm and 460 nm, respectively. K_D_ values were determined by plotting the free protease concentration as a function of the total inhibitor concentration. The experimental data were fitted with the following equation: y = E–(E+x+K–sqrt((E+x+K)^2^–4Ex))/2, where the independent variable x represents the total inhibitor concentration, the dependent variable y is the free protease concentration in equilibrium, K is K_D_, and E designates the total protease concentration.

## Results

### Phage display studies

**Figure 1:**
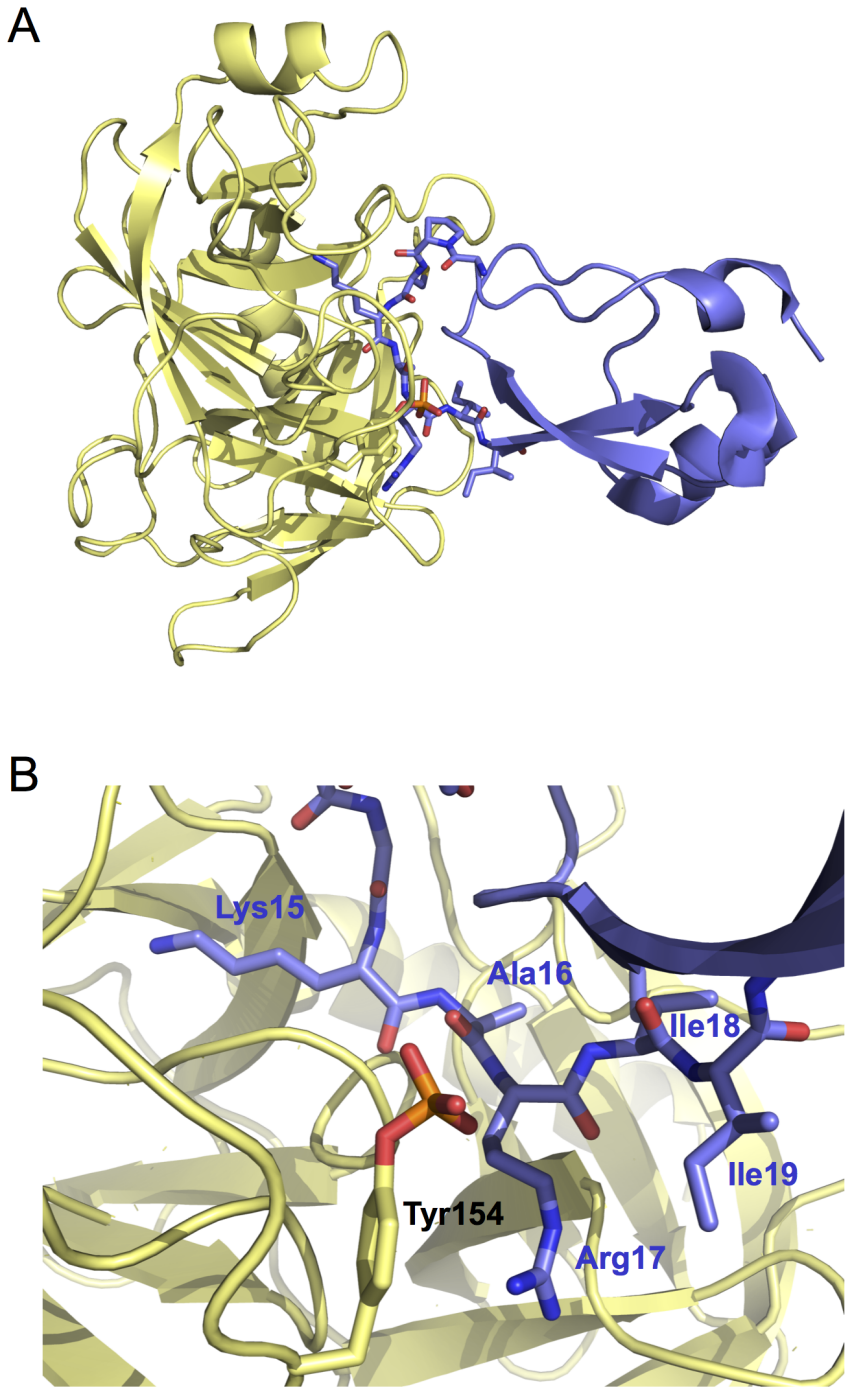
Modeled interaction of sulfated Tyr154 in human cationic trypsin with bound BPTI. (**A**) Ribbon diagram of sulfated cationic trypsin–BPTI complex. The model was created by fitting the sulfated trypsin (1TRN) on the structure of non-sulfated trypsin in complex with BPTI inhibitor (2RA3). (**B**) Prime side interactions of BPTI with sulfated cationic trypsin. The tyrosyl-sulfate (red) is within 2–4 Å of the P2′ Arg17 of BPTI.

To assess the potential impact of sulfation on substrate recognition by trypsin from a structural perspective, we fitted the crystal structure of native, sulfated cationic trypsin (Protein Data Bank code 1TRN) onto the structure of non-sulfated, recombinant cationic trypsin in complex with the trypsin inhibitor BPTI (Protein Data Bank code 2RA3) [5], [28]. As shown in Figure 1, this model indicates that the sulfated Tyr154 lies within 2–4 Å distance of the P2′ Arg17 side chain of BPTI. The position of Tyr154 suggests that sulfation might modify the selectivity of trypsin toward prime side residues of inhibitors and substrates; the P2′ position in particular.

**Figure 2:**
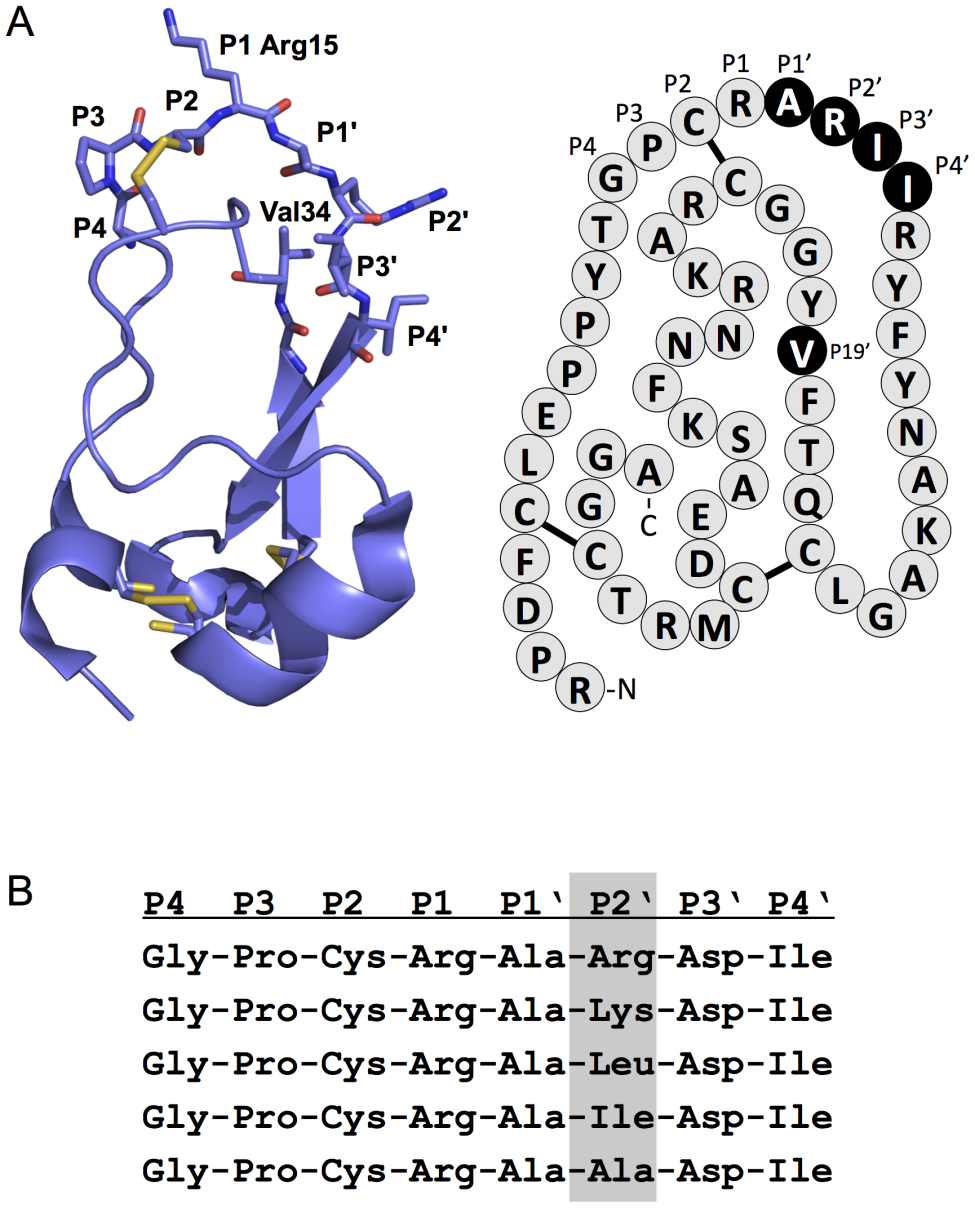
BPTI variants used for phage display and binding experiments. (**A**) Ribbon model and primary structure of K15R mutant BPTI indicating in black the randomized amino acid positions in the phage library. (**B**) Binding loop sequences of five BPTI variants designed to test the significance of the P2′ position. See text for details.

**Figure 3:**
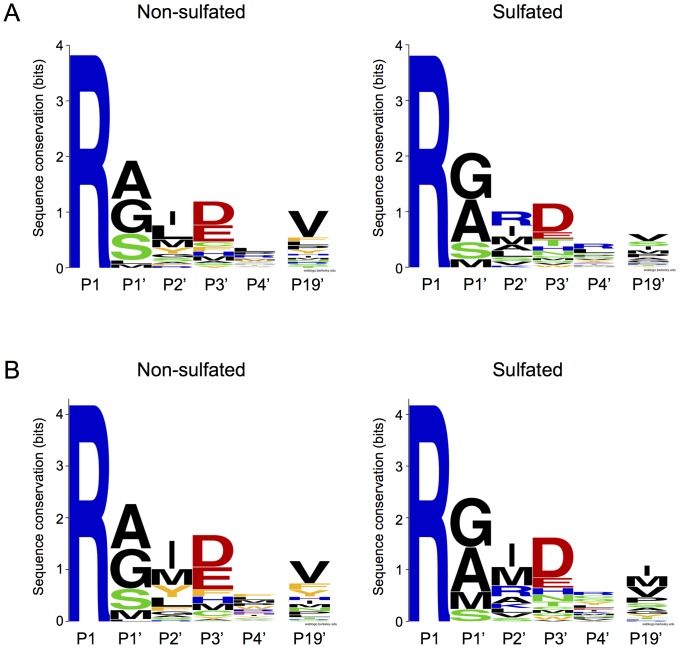
Sequence logo representation of phage display-selected BPTI variants against non-sulfated and sulfated trypsin. Randomized positions P1′, P2′, P3′, P4′ and P19′ are shown; the P1 position which was fixed as Arg is also included for reference. The overall height of the stack of symbols indicates the degree of sequence conservation at that position, whereas the height of symbols within the stack indicates the relative frequency of each amino acid. The colors indicate the chemical properties of the amino acid side chains; apolar is black, aromatic is orange, polar is green, acidic is red and basic is blue. The logos were created with the WebLogo program [27]. (**A**) Sequencing data from 28 and 27 independent BPTI clones selected after three panning cycles against non-sulfated and sulfated trypsins, respectively (Table 1). (**B**) Sequencing data was normalized to expected codon frequencies as described in *Experimental Procedures*.

To test this hypothesis, we designed a phage display library using BPTI as scaffold where the prime side amino acid residues in the reactive site loop, from P1′ through P4′, were fully randomized (Figure 2A). In addition, the P1 Lys residue was changed to Arg, and Val34 in the BPTI scaffold (P19′), which interacts with the P2′ position, was also randomized. Phages carrying tight binding BPTI inhibitors were selected against non-sulfated and sulfated human cationic trypsins. Following three selection cycles and verification by phage ELISA, selected clones were subjected to DNA sequencing. The overall selection pattern was highly similar for non-sulfated and sulfated trypsins, characterized by a slight-to-moderate preference for small side-chains at P1′ (Ala, Gly, Ser), mostly aliphatic amino-acids at P2′ (Ile, Met, Leu); acidic residues at P3′ (Asp, Glu) (Table 1 and Figure 3) and no selection at P4′. At position P19′ mostly the native Val was selected against non-sulfated trypsin, whereas a small enrichment for residues with short side chains (Ala, Gly, Ser) was detected in the selected clones against sulfated trypsin (Table 1).

**Table 1:** Amino acid sequence of the P1–P1′–P2′–P3′–P4′ and P19′ positions in 28 and 27 BPTI variants phage-display selected against non-sulfated (left panel) and sulfated (right panel) cationic trypsins, respectively.

Non-sulfated						Sulfated					
P1	P1′	P2′	P3′	P4′	P19′	P1	P1′	P2′	P3′	P4′	P19′
R	A	M	C	P	M	R	G	**R**	D	Q	M
R	L	I	A	P	V	R	G	M	H	A	V
R	S	I	E	I	V	R	S	I	D	Y	A
R	G	I	D	L	V	R	A	K	T	V	S
R	A	I	E	W	V	R	A	I	N	N	P
R	S	I	S	R	I	R	S	**R**	T	Y	A
R	G	L	E	F	P	R	G	M	T	R	I
R	S	I	D	W	V	R	A	M	E	P	V
R	A	L	D	C	L	R	A	A	E	R	G
R	A	G	M	M	L	R	A	I	D	L	R
R	S	L	D	Y	P	R	S	L	N	L	I
R	G	L	D	V	Y	R	G	L	E	P	M
R	G	**R**	H	F	S	R	A	L	D	R	G
R	A	I	D	L	V	R	G	**R**	Q	C	V
R	G	S	M	L	F	R	A	I	D	R	V
R	A	L	C	S	F	R	A	**R**	D	M	S
R	G	A	H	L	Y	R	G	A	D	Q	V
R	A	L	D	R	V	R	M	I	W	L	S
R	G	Y	F	A	V	R	G	**R**	C	L	S
R	A	M	D	P	V	R	A	**R**	D	I	P
R	A	M	E	H	N	R	A	S	D	R	L
R	G	Y	D	R	V	R	G	M	D	C	I
R	M	G	E	P	V	R	G	S	M	E	Y
R	S	S	E	R	R	R	G	A	H	K	P
R	G	Y	Y	M	T	R	M	**R**	D	V	M
R	S	M	D	L	V	R	G	V	D	L	T
R	S	F	F	L	V	R	S	V	V	R	V
R	A	L	E	E	H						

These phage clones differed at the DNA sequence level, indicating independent selection. Arg residues selected at P2′ are highlighted in bold and underlined.

The most significant difference in the selection pattern between non-sulfated and sulfated trypsins was at the P2′ position, where Arg was selected against sulfated trypsin with the highest frequency (seven occurrences), whereas only one clone selected against non-sulfated trypsin carried a P2′ Arg (Table 1). However, when the selected sequences were normalized to reflect the expected codon frequencies in the phage library, the P2′ Arg preference became less significant (Figure 3B). To exclude the possibility that a display bias in the BPTI library might account for the observed selection pattern, a panning cycle was performed against immobilized anti-BPTI IgG. One hundred independent ELISA-positive clones were sequenced and all investigated positions in the library were found fully random. Taken together, the phage display selection results indicate that sulfation of cationic trypsin increases selectivity towards P2′ Arg versus hydrophobic P2′ side chains.

### BPTI binding studies

**Figure 4:**
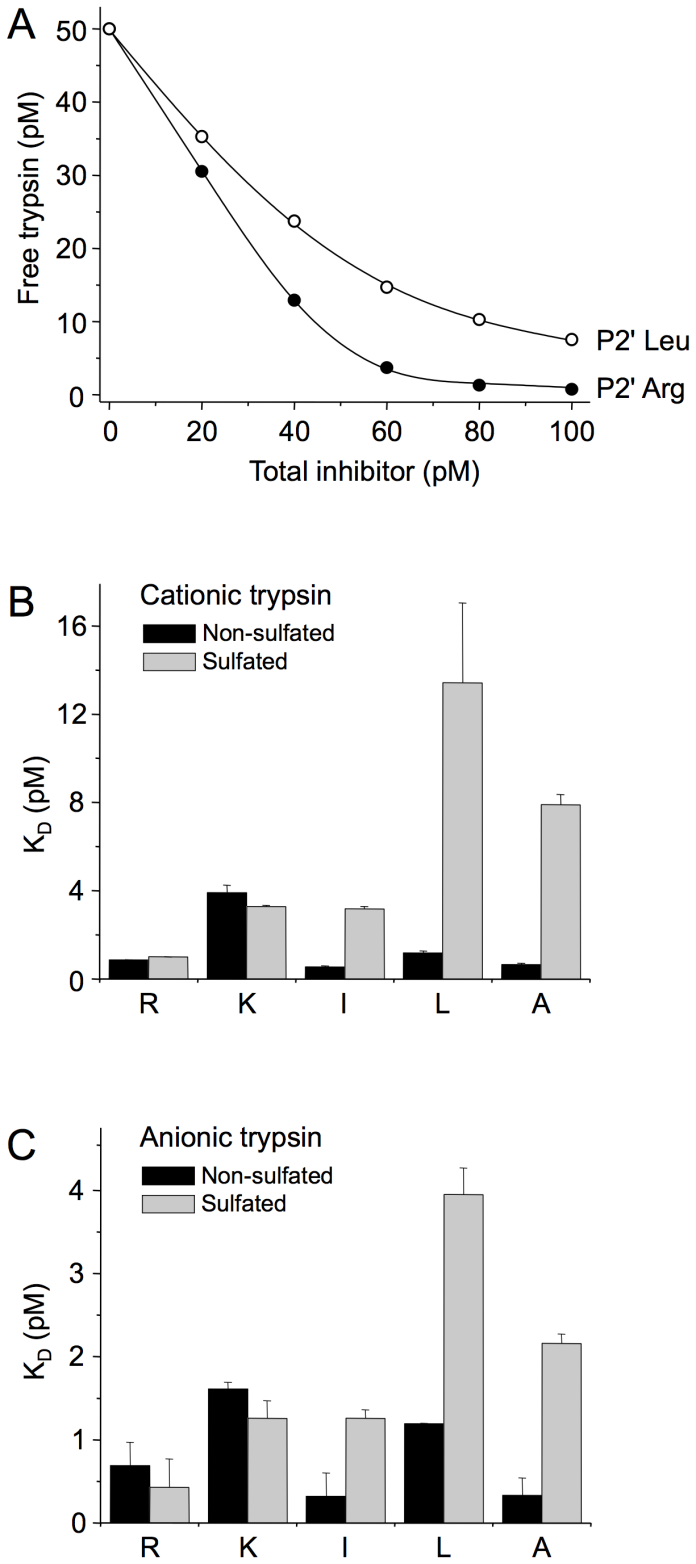
Inhibition of non-sulfated and sulfated trypsin by phage-display selected BPTI variants. (**A**) Representative inhibitor binding experiments are shown using sulfated cationic trypsin and BPTI variants with a P2′ Arg and Leu. See *Experimental Procedures* for details. (**B**) The effect of the P2′ amino acid in BPTI on the inhibition of non-sulfated (black bars) and sulfated (gray bars) human cationic trypsin. K_D_ values from Table 2 were plotted. (**C**) The effect of the P2′ amino acid in BPTI on the inhibition of non-sulfated (black bars) and sulfated (gray bars) human anionic trypsin. K_D_ values from Table 2 were graphed.

To test the significance of the P2′ position in a more direct fashion, we designed individual BPTI variants carrying Arg, Lys, Leu, Ile or Ala at P2′ (Figure 2). On the basis of the phage display selection pattern, positions P1′, P3′, P4′ and P19′ were fixed as Ala, Asp, Ile, and Val, respectively. The five BPTI variants were expressed in *Pichia pastoris* and purified to homogeneity. To characterize BPTI binding to non-sulfated and sulfated cationic trypsins, we performed equilibrium binding assays and determined the equilibrium dissociation constants (K_D_) (Figure 4A). All inhibitor variants exhibited high affinity (K_D_ ranged form 0.5 pM to 13 pM). Variants with Arg or Lys at P2′ bound to non-sulfated and sulfated cationic trypsins with unchanged affinities; however, variants harboring apolar residues (Ile, Leu or Ala) at P2′ exhibited 6–12-fold weaker affinity to sulfated trypsin relative to the non-sulfated enzyme (Table 2, Figure 4B). To confirm and extend these observations, the BPTI variants were also tested against non-sulfated and sulfated human anionic trypsins. Again, equilibrium dissociation constant values indicated high affinity binding (K_D_ range 0.3–4 pM) (Table 2). Similarly to our findings with cationic trypsin, no difference in binding to non-sulfated and sulfated anionic trypsins was found with variants carrying Arg or Lys at P2′, whereas inhibitors containing a hydrophobic P2′ residue bound to sulfated anionic trypsin with approximately 3–7-fold weaker affinity compared to non-sulfated anionic trypsin (Table 2, Figure 4C).

**Table 2:** Inhibition of non-sulfated and sulfated cationic and anionic trypsins by phage display-selected BPTI variants.

	BPTI P2′	Non-sulfated	Sulfated
	R	0.9±0.1	1.0±0.1
	K	3.9±0.4	3.3±0.1
Cationic trypsin	L	1.2±0.1	13.4±3.6
	I	0.5±0.1	3.2±0.1
	A	0.7±0.1	7.9±0.5
	R	0.7±0.3	0.4±0.3
	K	1.6±0.1	1.3±0.2
Anionic trypsin	L	1.2±0.1	4.0±0.3
	I	0.3±0.2	1.3±0.1
	A	0.3±0.2	2.2±0.1

Equilibrium dissociation constants (K_D_) were determined as described in *Experimental Procedures* and values were expressed in picomolar units of concentration. Averages ± SEM from two independent K_D_ determinations are indicated. Note that each K_D_ value was determined from five independent binding events (see Figure 4A) and each binding event was assayed in duplicate.

When the averaged K_D_ values for BPTI variants with apolar and basic P2′ residues are considered, a small, 2–3-fold selectivity for apolar versus basic P2′ residues is evident in non-sulfated trypsin (0.8 pM versus 2.4 pM in cationic trypsin and 0.6 pM versus 1.2 pM in anionic trypsin). This trend becomes reversed in sulfated trypsin, which exhibits a 3–4-fold preference for basic P2′ residues over hydrophobic side chains (8.2 pM versus 2.2 pM in cationic trypsin and 2.5 pM versus 0.9 pM in anionic trypsin). When the same calculations are performed for Arg versus all apolar P2′ side chains, no selectivity is observed with non-sulfated trypsin, whereas sulfated trypsin prefers Arg over hydrophobic P2′ amino acids by a factor of 6–8. Thus, the net effect of tyrosine sulfation on human trypsins is an increase in P2′ selectivity towards basic residues versus hydrophobic residues by about an order of magnitude.

## Discussion

In the present study we used phage display technology to investigate the significance of tyrosine sulfation in the substrate binding specificity of human cationic and anionic trypsins. Our findings demonstrate that the negatively charged sulfate group on Tyr154 modifies the P2′ selectivity of trypsins; it slightly inhibits binding of hydrophobic side chains (Ala, Ile, Leu), whereas it maintains an essentially unaltered affinity for positively charged Arg and Lys residues. The overall effect of sulfation is a 6–12-fold increase in selectivity towards basic P2′ residues. This conclusion is consistent with structural modeling showing steric proximity between Tyr154 and the P2′ side chain of bound inhibitor (see Figure 1).

We also found that human cationic trypsin favored residues with short side chains (Ala, Gly, or Ser) at P1′ and acidic residues (Asp or Glu) at P3′; these selection patterns were independent of sulfation. These observations differ from previous studies mapping the S1′ subsite of bovine and rat trypsins using acyl-transfer experiments, which found that the S1′ site exhibited broad specificity with an apparent preference toward hydrophobic side chains rather than Ala/Gly/Ser as observed here [29]–[33]. Similarly broad specificity without selectivity for Asp/Glu was observed for the S3′ site in rat trypsin [30]. The different selection pattern in our experiments may be related to several differences between the acyl-transfer experiments and the phage display approach taken here. An important difference is that preferences revealed in acyl-transfer experiments encompass both binding affinity and catalytic competence for ligation; a successful nucleophile must not only bind to the prime side subsites of the enzyme, but also carry out productive nucleophilic attack on a second substrate occupying the nonprime side subsites of the enzyme. By contrast, phage display selection unmasks the binding preferences of the enzyme uncoupled from catalytic rates. Another contributing factor may be the conformational constraints of the relatively rigid BPTI scaffold. Indeed, mutational analysis of the P1′ position of BPTI found Ala, Gly and Ser as the preferred residues for tight binding to trypsin, chymotrypsin and plasmin [34]. Of interest, another study mapping binding preferences of bovine trypsin using fluorescence-quenched substrates found a pattern of specificity more closely approximating our results, in which Ser and Ala (in addition to Arg) were preferred residues at P1′, and Asp was among the more favored residues at P3′ [35].

The P1′ and P3′ specificities observed may be interrelated, as these subsites often have a cooperative relationship. Owing to the extended (canonical) backbone conformation of bound inhibitors and substrates, side chains at the P1′ and P3′ positions point in the same direction and their interactions with the S1′ and S3′ subsites are contiguous. Crystal structures indicate that Lys66 (corresponding to Lys60 in conventional chymotrypsin numbering) may be a determinant of S1′ and S3′ specificity [36], and the Asp/Glu selection at P3′ may be explained by a favorable electrostatic interaction. This interaction, in turn, may partially obstruct the S1′ subsite, resulting in the selection of small volume side chains at P1′ instead of larger hydrophobic residues. A similarly restricted P1′ preference is also characteristic of thrombin, where Lys60f (chymotrypsin numbering) occludes the S1′ subsite and limits its specificity to amino-acids with small side-chains [37], [38].

The biological significance of the observed P2′ selectivity for basic amino acids in sulfated human trypsins remains unclear. As detailed in the introduction, previous studies were unable to identify a convincing role for tyrosine sulfation in trypsin function. The large majority of vertebrate trypsins do not appear to be sulfated, as judged by the absence of Tyr154 or the required sulfation motif (see Table 1 in reference [6]). This suggests that trypsin sulfation in humans may have evolved to facilitate the digestion of specialized substrates present in the primate diet only. Alternatively, the true evolutionary driving force of trypsin sulfation may have been unrelated to catalytic activity and the relatively small changes in substrate specificity may be inconsequential in the digestive function of trypsins. Yet a third possibility is that sulfation may enhance the catalytic capability of trypsins toward protein substrates by weakening prime side interactions. For protein substrates, strong affinity between the prime side residues and corresponding protease subsites will have the effect of retarding the deacylation step of the reaction. For good substrates of trypsin, deacylation can be the rate determining step in the overall reaction [39], [40]. As a consequence, by diminishing prime side affinity for the majority of protein substrates the effect of Tyr154 sulfation may be to accelerate enzyme turnover. Interestingly, rat anionic trypsin-2 contains a Glu residue in place of Tyr154 and this negatively charged side chain may mimic the function of a sulfated Tyr154. Indeed, a salt bridge formed between Glu154 and the P2′ Arg of the bound inhibitor is evident in the crystal structure of rat anionic trypsin-2 with BPTI [36]. Furthermore, prime side mapping of rat trypsin using acyl-transfer experiments demonstrated an S2′ preference for positively charged residues [30].

In summary, we demonstrated that sulfation of human anionic and cationic trypsins on Tyr154 increases selectivity towards basic versus apolar residues at the P2′ position of inhibitors that bind in a substrate-like fashion. Although the increase in selectivity is relatively small, we speculate that this post-translational change in substrate specificity may facilitate digestion of a broader range of dietary proteins.
